# Extraction of Fungal Chitosan by Leveraging Pineapple Peel Substrate for Sustainable Biopolymer Production

**DOI:** 10.3390/polym16172455

**Published:** 2024-08-29

**Authors:** Delwin Davis, Mridul Umesh, Adhithya Sankar Santhosh, Sreehari Suresh, Sabarathinam Shanmugam, Timo Kikas

**Affiliations:** 1 Department of Life Sciences, CHRIST (Deemed to be University), Hosur Road, Bengaluru 560029, Karnataka, India; 2 Institute of Forestry and Engineering, Estonian University of Life Sciences, Kreutzwaldi 56, 51014 Tartu, Estonia

**Keywords:** agro-waste, fungal chitosan, pineapple peel waste, biopolymer production, food packaging

## Abstract

The cost-effective production of commercially important biopolymers, such as chitosan, has gained momentum in recent decades owing to its versatile material properties. The seasonal variability in the availability of crustacean waste and fish waste, routinely used for chitosan extraction, has triggered a focus on fungal chitosan as a sustainable alternative. This study demonstrates a cost-effective strategy for cultivating an endophytic fungus isolated from Pichavaram mangrove soil in a pineapple peel-based medium for harvesting fungal biomass. Chitosan was extracted using alkali and acid treatment methods from various combinations of media. The highest chitosan yield (139 ± 0.25 mg/L) was obtained from the pineapple peel waste-derived medium supplemented with peptone. The extracted polymer was characterized by FTIR, XRD, DSC, and TGA analysis. The antioxidant activity of the fungal chitosan was evaluated using DPPH assay and showed an IC_50_ value of 0.22 mg/L. Subsequently, a transparent chitosan film was fabricated using the extracted fungal chitosan, and its biodegradability was assessed using a soil burial test for 50 days. Biodegradation tests revealed that, after 50 days, a degradation rate of 28.92 ± 0.75% (*w*/*w*) was recorded. Thus, this study emphasizes a cost-effective strategy for the production of biopolymers with significant antioxidant activity, which may have promising applications in food packaging if additional investigations are carried out in the future.

## 1. Introduction

Food waste is a significant challenge faced by the agricultural sector in this century. One-third of the total food produced is usually wasted and ends up as food waste, creating different types of environmental issues, such as the production of offensive odors, water and soil pollution, and serving as a source of pathogens. Fruits, vegetables, and roots contribute to half of this global waste that ends up in landfills. Their decomposition produces poisonous gases, such as methane, leading to 7% of greenhouse emissions [1]. To address this issue, value-added products such as biopolymers and biofuels are produced, and many bioactive compounds such as enzymes, pigments, and polysaccharides are extracted from waste materials [2]. Pineapple (*Ananas comosus*) is the third most important tropical fruit, with more byproducts than subtropical and temperate fruits, and is mainly used for juice, pulp, jam, and jellies. Pineapple production is increasing by 1.9% annually, and is expected to reach approximately 31 million tons by 2028. Only half the weight of the pineapple is utilized; the remaining pineapple peel, core, pulp, pomace, and leaf are disposed of as waste [3]. Various studies have shown that pineapple waste contains different potential raw materials, such as insoluble fibers, pectin, simple sugars, proteins, vitamins, minerals, and phenolic compounds. It has been reported that pineapple waste includes 82% sugar, of which 55% is reducing and 27% is non-reducing [4]. Numerous studies have focused on the use of pineapple waste as a raw material to produce various value-added products, such as fibers, organic acids, and phenolic compounds. Bromelain is an effective protease enzyme that is exclusively extracted from pineapple waste and has numerous therapeutic and pharmacological applications [5]. Biodiesel and bioethanol have been generated because pineapple wastes are rich in carbohydrates and sugars to replace the fossil fuel-oriented generation with sustainable ones. Owing to their antioxidant properties, pineapple byproducts can be utilized in the food industry to prevent oxidative degradation of fatty foods and fruity aroma [6].

Chitin is a natural, cationic, and non-toxic biopolymer, and is the second most abundant biopolymer after cellulose [7]. Chitin biopolymers are present in high quantities in fungal cell walls, arthropod exoskeletons, and fish scales. The cationic nature of chitin is attributed to the presence of a quaternary functional salt group. Chitosan is a D-glucosamine polymer produced by the deacetylation of chitin. Chitosan has various pharmaceutical, therapeutic, and cosmeceutical applications because of its unique antibacterial, antifungal, and adsorption properties [8]. Chitosan has also been reported as a carrier for drug delivery. Research confirms that chitosan carriers shield and preserve 75% of the medication from breaking down due to water exposure while maintaining its effectiveness. Additionally, chitosan has been applied in crafting bandages and materials for wound dressings owing to its wound-healing abilities, pain-relieving effects, and anti-itch properties [9]. Chitosan is used in the food industry to increase the shelf-life of fruits, vegetables, meat, and fish [10]. Chitosan-coated fish fillets and meat slices showed retarded lipid oxidation and microbial growth compared with untreated ones. Chitosan can be used as an active packaging material because of its barrier, optical, and mechanical properties. The versatile material properties of chitosan make it a sustainable alternative to synthetic plastics if critical research attention is to be focused on this domain [11].

Crustacean shells are primarily utilized for chitosan production, which requires intense acid and alkali treatments and is hazardous to the environment. Chitin extraction (for chitosan preparation) from crustacean shells involves demineralization using acid treatment to remove the CaCO_3_ in the shell, followed by deproteinization by reacting with aqueous alkali (1–10% NaOH, at 65–100 °C) to remove the protein and organic components (other than chitin). Deproteinization followed by demineralization is reported to be equally effective in extracting chitin from chitosan. The extracted chitin is then deacetylated by treating it with heated alkali (40–50%) to produce chitosan [12]. Variations in the quantity and quality of crustacean shell waste available for chitosan extraction serve as a major drawback for its use in a commercial chitosan production process, and the chitosan yield may also be affected by the seasonal availability of the substrate. The use of fungi for chitosan extraction can address this issue, as a stable chitosan yield can be achieved through optimized protocols.

Polysaccharides, such as chitin and glucan, are structural components of fungal mycelial cell walls. The interstitial components of fungal cell walls are composed of glycoproteins such as mannoproteins, galactoproteins, xylomannoproteins, and glucuronoproteins [13]. It is reported that chitin constitutes 45% of the cell wall of *Aspergillus niger* [14]. Fungal chitin can be converted to chitosan through a deacetylation process [15]. Chitosan is soluble in acids including acetic acid and lactic acid, and its solubility is highly dependent on its molecular weight and degree of deacetylation. The lower ash content in fungal chitin eliminates the demineralization step required for crustacean shells during chitin and chitosan extraction [16]. Because fungal chitin has a lower ash content than crustacean shell waste, demineralization is not required during processing.

Fungal mycelia also lack the allergic protein tropomyosin and various inorganic compounds, making them an ideal choice for chitosan extraction [17]. Pineapple peel is a rich source of insoluble fibers, mainly cellulose, which accounts for 20–25% of the dry weight. Microorganisms can hydrolyze it and use it as an energy source [3]. Pineapple crowns also contain high amounts of cellulose (79–83%), hemicellulose (19%), and lignin (5–15%) and have been utilized for the growth of microorganisms [3]. This study focused on developing a sustainable strategy for chitosan extraction from a fungal biomass cultivated in a pineapple peel waste-based medium to serve as a cost-effective alternative to the synthetic media used for fungal cultivation. The extracted fungal chitosan was harnessed to create a biodegradable sheet with notable antioxidant properties that can be utilized as a food packaging material to bolster food safety measures.

## 2. Materials and Methods

### 2.1. Media and Chemicals

All media components for the current study were procured from HiMedia Laboratories Pvt., Ltd. (Mumbai, India). Analytical grade (AR) solvents used in the current study were purchased from Merck (Darmstadt, Germany) [18].

### 2.2. Fungal Biomass Production

#### 2.2.1. Biomass Production Using Synthetic Media

Sabouraud dextrose broth (SDB) (100 mL) was prepared and sterilized by autoclaving at 121 °C for 30 min. After cooling to room temperature, a single fungal inoculum disc of DEL01 was inoculated into liquid broth (detailed information of the fungal isolates labelled DEL01–DEL10 [19,20,21,22,23] and the molecular identification [24,25] is described in the Supporting Information) [26]. The mixture was then subjected to static incubation for 7 days at room temperature for biomass growth [27].

#### 2.2.2. Biomass Production of Pineapple Peel Waste

Pineapple peel waste was collected from the KR Market in Bangalore, India. It was thoroughly washed with distilled water to eliminate dirt and then air-dried at room temperature to reduce the excess moisture content. Following drying, the waste was blended with distilled water and the liquid portion was extracted. Three different media combinations were prepared: J + P + D [pineapple peel juice (100 mL) + peptone (10 g/L) + dextrose (40 g/L)], J [pineapple peel juice (100 mL)], and J + P [pineapple peel juice (100 mL) + peptone (10 g/L)]. The components were mixed thoroughly in a 250 mL Erlenmeyer flask, autoclaved at 121 °C for 30 min, and cooled to room temperature. Fungal inoculum discs were added and incubated for 7 d at 30 °C [28].

### 2.3. Extraction of Fungal Chitosan

The fungal biomass, aged 7 days, was rinsed with distilled water to eliminate any residual media content. Subsequently, the mixture was treated with 1 M NaOH solution (1:40) and autoclaved at 121 °C for 15 min. Alkali treatment was intended to remove protein and undesired substances. The mixture was then centrifuged at 10,000 rpm for 15 min, and the alkali-insoluble pellet was separated and washed to neutralize the pH. The next phase was acid treatment, in which demineralization occurred. The alkali-insoluble fungal fraction was treated with 2% acetic acid (1:30) for 8 h at 95 °C in a water bath to dissolve fungal chitosan. The mixture was filtered, and the filtrate pH was adjusted to 10 to precipitate the dissolved fungal chitosan. The precipitated chitosan was then washed with water, ethanol, and acetone. The obtained chitosan was dried at 60 °C and the yield was calculated [17].

### 2.4. Characterization of Extracted Chitosan

The extracted chitosan was characterized using Fourier transform infrared spectroscopy (FTIR), X-ray diffraction (XRD), thermogravimetric analysis (TGA), and differential scanning calorimetry (DSC). FTIR spectroscopy was used to analyze the IR bands specific to the functional groups present in the chitosan biopolymer. Fungal chitosan was scanned from 4000 to 400 cm^−1^ at a resolution of 4 cm^−1^. Fourier transform infrared (FTIR) analysis was performed using a Shimadzu IR Spirit-L FTIR spectrophotometer. XRD analysis determined the angular diffraction (2*θ*) pattern of the biopolymer between 5° and 90° to elucidate the crystal phase composition, structure, and orientation of the extracted fungal chitosan [7]. The MiniFlex 600 XRD (Rigaku, Tokyo, Japan) at the Central Instrumentation Facilities, CHRIST (Deemed to be University), Bangalore, was utilized to perform the XRD analysis in this study. The crystallinity index of chitosan was determined by analyzing the area under the XRD peaks. TGA assesses the thermal stability of biopolymers by constantly monitoring mass changes with increasing temperatures [29]. TGA was performed using 10 mg of the extracted chitosan at a heating rate of 20 °C/min from 40 °C to 800 °C [SDT Q600 V20.9 Build 20, Thermal Gravimetric Analyzer, (TA Instruments, New Castle, DE, USA)]. DSC analysis provides insight into material properties, such as the glass transition range, crystallization, and melting point of the extracted biopolymer. DSC analysis of the extracted chitosan (10 mg) was performed using a Perkin Elmer STA 6000 (Walthan, MA, USA) [30].

### 2.5. Antioxidant Activity of Extracted Chitosan

The antioxidant activity of the extracted chitosan was assessed to widen its application in various fields, such as food packaging and food additives. Antioxidant activity was measured using a 2,2-diphenyl-1-picrylhydrazyl (DPPH) free radical scavenging assay. Different concentrations (0.25 mg to 2 mg) of fungal chitosan were prepared by dissolving chitosan in 1% acetic acid. To these solutions, 5 mL of 100 μmol/L DPPH methanol was added and incubated at room temperature in the dark for 30 min. Absorbance was measured after incubation at 517 nm using a SHIMADZU UV spectrophotometer (UV-1800, Kyoto, Japan). All readings were taken in triplicate. The percentage inhibition of free radicals by the sample was calculated using the following formula, where *A*_0_ is the absorbance of the blank solution and *A*_1_ is the absorbance of the reaction solution:

$$Percentage\;Inhibition=\left(\frac{A_{0}-A_{1}}{A_{0}}\right)\times 100$$

### 2.6. Fabrication of Chitosan Film

A thin film of chitosan was fabricated by adding extracted chitosan (0.5 g) to 50 mL of 1% (*v*/*v*) acetic acid. The solution was then heated at 60 °C and 650 rpm for 3 h. From this solution, 20 mL was poured into a petri dish and allowed to dry at 55 °C for 6 h to form a thin transparent chitosan film [31].

### 2.7. Biodegradation Study of the Chitosan Film

The biodegradability of the fabricated chitosan films was tested using the soil burial method. The films were cut into dimensions of 2 cm × 2 cm and wrapped in a thin non-degradable plastic mesh. The initial weight of each setup was measured before burying in 1 kg of garden soil at a depth of 1 cm within a plastic tray. The entire biodegradation apparatus was maintained at room temperature with 60% moisture level. Weight reduction was measured every 10 days, and the percentage degradation of the film was measured using the following formula:

$$Degradation\;percentage\;(\%)\;(w/w\%)=\left(\frac{Initial\;weight-Final\;weight}{Initial\;weight}\right)\times 100$$

## 3. Results and Discussion

### 3.1. Extraction of Chitosan from Fungal Biomass

**Figure 1:**
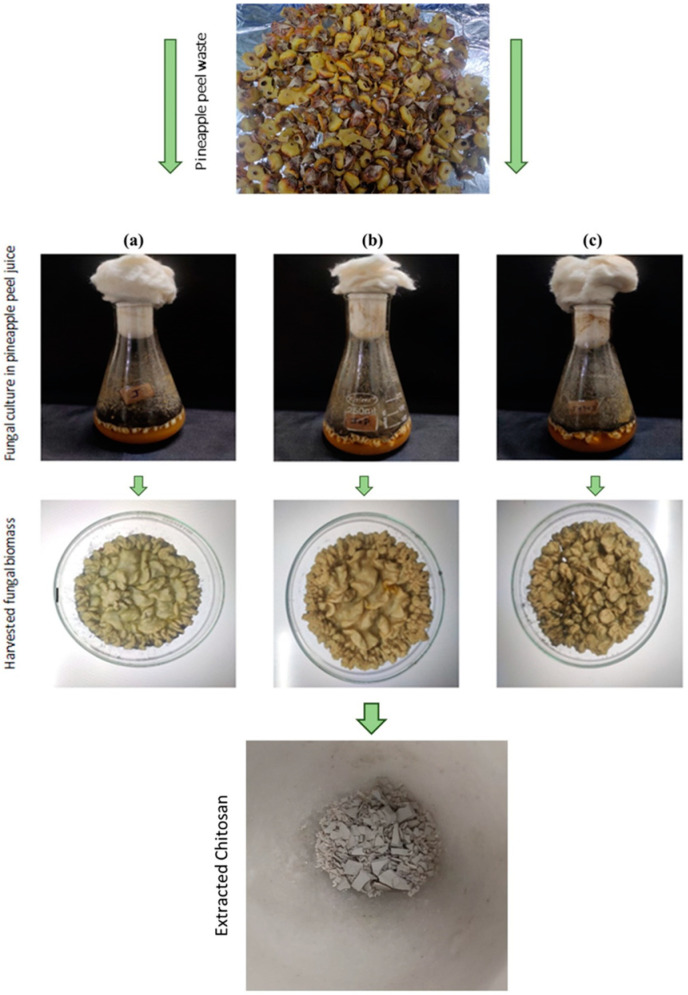
(**a**) Fungal biomass from pineapple peel media, (**b**) fungal biomass from pineapple peel media with peptone, and (**c**) fungal biomass from pineapple peel media with peptone and dextrose.

After 7 days of incubation, the fungal chitosan was extracted (Figure 1). The results indicated that the highest chitosan yield was observed for J + P, with a value of 139 ± 0.25 mg/L. J + P + D exhibited a yield of 98 ± 0.33 mg/L. The lowest yield was recorded for J at 63.4 ± 0.17 mg/L. The growth observed in the pineapple waste substrate significantly surpassed that of the synthetic SDB medium, which yielded 24 ± 0.8 mg/L of chitosan. The obtained results (Table 1) suggest that pineapple peel extract serves as an ideal medium for the culture of *Aspergillus niger*, providing essential sugar and nitrogen sources necessary for optimal growth and multiplication [32]. In setup J, a significant amount of sugar, which is naturally present in pineapple, served as the primary nutrient source. However, a substantial increase in fungal biomass was observed when peptone was added as the nitrogen source to the J + P media. The yield in the J + P + D setup might have been comparatively lower, potentially because of the high sugar content, which could affect the growth dynamics of the fungus. Rasmussen et al. [33] reported that fungal growth declined by 50% with high sugar content and 40% with high nitrogen content.

Fungal chitosan production using banana peel waste medium under optimized conditions from *Aspergillus niger* (accession number ON53364) was reported to be 200 mg/L, which was similar to the highest yield obtained in the current study [34]. Abasian et al. [34] reported a chitosan yield of 70.3 mg/L from *M. rouxii* cultivated in media containing glucose and fungal extracts. Namboodiri et al. [35] reported a maximum chitosan yield of 170 mg/L in paper mill effluent for chitosan production from *Penicillium Citrinum* under continuous mode operation in a bioreactor.

### 3.2. Characterization of Extracted Chitosan

**Figure 2:**
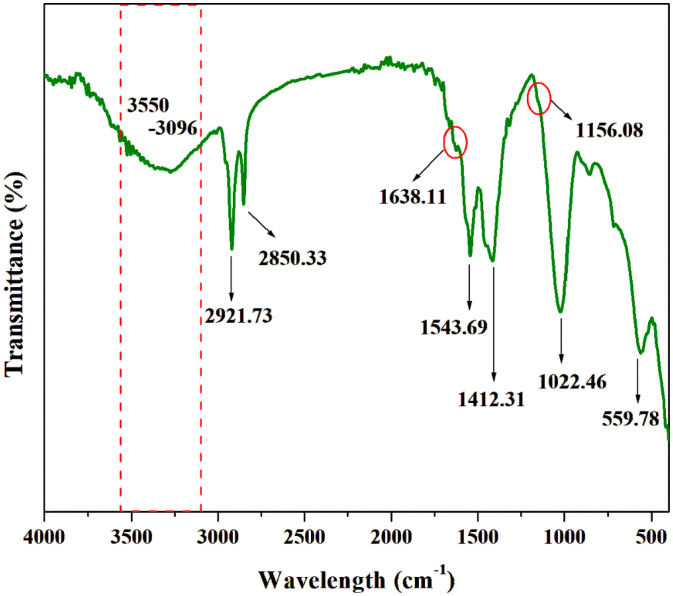
FTIR spectrum of the extracted fungal chitosan.

FTIR, XRD, TGA, and DSC were used to characterize the chitosan extracted from J + P (the combination that showed the highest yield). FTIR spectra obtained for the fungal chitosan (Figure 2) aligned with the standard chitosan peaks of absorption. The O–H stretching (hydroxyl groups in chitosan) and N–H stretching vibrations overlapped, forming a broader peak between 3550 and 3096 cm^−1^ [36]. The sharp absorption peaks at 2921.73 and 2850.33 cm^−1^ were attributed to the stretching of C-H bonds in CH_3_ groups [37]. The shoulder peak at 1638.11 cm^−1^ was assigned to the N–H bending vibration of the primary amines in chitosan. The characteristic peak at 1543.69 cm^−1^ confirmed the presence of N–H bending vibrations of amide II, whereas the peak at 1412.31 cm^−1^ was ascribed to O–H bending vibrations of the extracted fungal chitosan [38]. The weak shoulder peak at 1156.08 cm^−1^ indicated the C–O–C stretching vibration associated with the saccharide ring structure, confirming glycosidic linkages in the fungal chitosan [39]. The peak at 1022.46 cm^−1^ indicated the C–O stretching vibration of the glucosamine ring in chitosan [40]. Additionally, the N–H bending vibrations were represented by the peak at 559.78 cm^−1^. The absence of peaks corresponding to common impurities suggested that the extracted chitosan was very pure.

**Figure 3:**
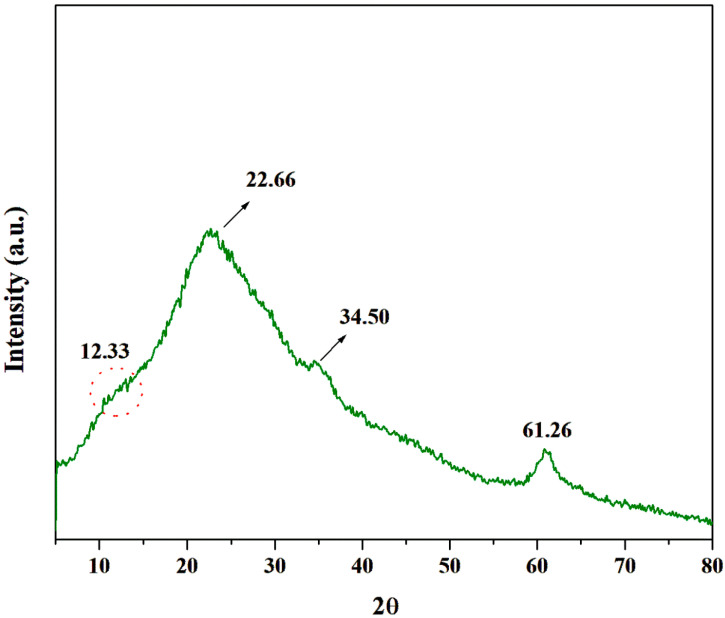
XRD spectrum of the extracted fungal chitosan.

The XRD spectrum of chitosan extracted from the fungal biomass is shown in Figure 3. The 2*θ* values of 12.33° and 22.66° were characteristic of chitosan [41]. The shoulder peak observed at 2*θ* = 12.33° represented the amorphous phase of chitosan (020) and the peak at 2*θ* = 22.66° corresponded to the crystalline phase of chitosan (110) [42]. Peaks that are often associated with specific crystallographic planes present in chitosan materials were observed at 2*θ* = 34.50° (002) and 2*θ* = 61.26°, which are less commonly reported and studied [43]. The crystallinity index (CI) of the extracted fungal chitosan was calculated to be 51.61%. In a similar study conducted by Kaya et al. [44], the CI of chitosan extracted from the medicinal fungus *Fomitopsis pinicola* was reported to be 52%. The chitosan extracted by Ssekatawa et al. [45] from various sources, including edible Ugandan mushrooms for biomedical applications, exhibited a CI of 48.4  ±  0.44%.

**Figure 4:**
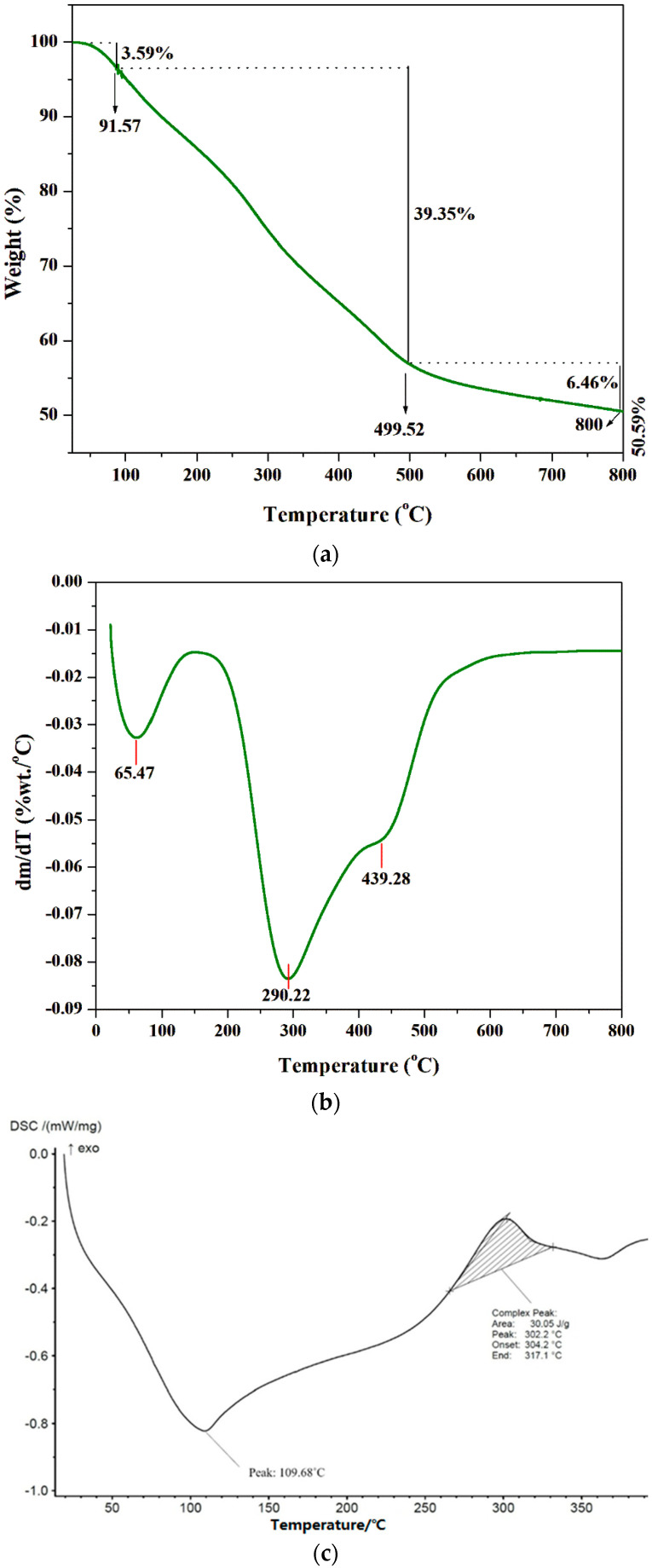
(**a**) TGA, (**b**) DTA, and (**c**) DSC curve of extracted fungal chitosan.

TGA, DTA, and DSC are powerful analytical tools for monitoring the physicochemical properties of polymers at various temperatures. The thermal stability of biologically extracted chitosan was analyzed using TGA (Figure 4a). The fungal chitosan exhibited an initial weight reduction of 3.59% (*w*/*w*) between 0 °C and 91.57 °C, which can be attributed to the removal of water molecules [46]. Between 91.57 °C and 499.52 °C, a single-stage weight loss of 39.35% (*w*/*w*) was observed, which can be related to the fast pyrolysis of the chitosan material and the disintegration of acetylated chitin groups [47]. However, in the DTG curve (Figure 4b), two distinct stages were observed between 100 °C and 500 °C, with peaks at 290.22 °C and 439.28 °C, corresponding to the depolymerization and degradation of the chitosan structure. Slow pyrolysis, resulting in a weight reduction of 6.46% (*w*/*w*), was observed between 499.52 °C and 800 °C, which can be associated with the deterioration of the pyranose rings [48]. The 50.59% (*w*/*w*) residue remaining after heating to 800 °C consisted of ash material and carbon, a product of the thermal degradation of chitosan [49]. This observation indicated the high-temperature stability of the extracted chitosan, as it can withstand elevated temperatures without undergoing rapid degradation.

The DSC curves of chitosan are shown in Figure 4c. The extracted chitosan was analyzed by gradually heating 10 mg of the polymer from room temperature to 400 °C at a rate of 10 °C/min [50]. The thermogram revealed a two-stage degradation process. The initial decomposition appeared as a broad endothermic peak around 109.68 °C, attributed to the loss of water molecules [51]. The second stage exhibited an exothermic peak between 265 and 330 °C, with a maximum at 302.2 °C, corresponding to the breakdown of the saccharide structure in the extracted chitosan [52]. These results confirmed the purity of the extracted fungal chitosan, as it demonstrated only two-stage degradation, similar to the commercial chitosan reported by Soon et al. [53].

### 3.3. Antioxidant Activity of the Extracted Chitosan

**Figure 5:**
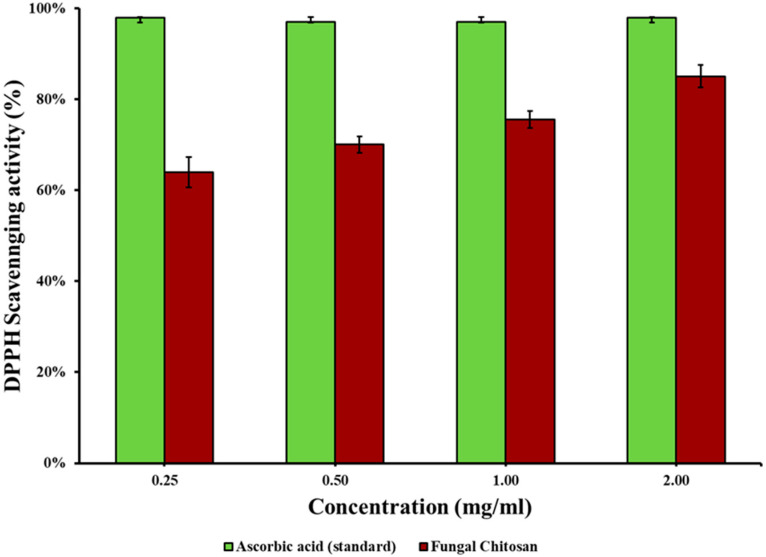
DPPH scavenging activity of the extracted fungal chitosan.

The DPPH free radical scavenging assay is the most commonly used method to determine antioxidant activity. Chemically, DPPH is a free radical that reacts with hydrogen radicals or free electrons to form stable diamagnetic molecules in association with a color change. Understanding the antioxidant activity of a biopolymer enhances its potential applications as an active food packaging material, which adds to the packaging application with preservation of packed foods [54]. The antioxidant activity of chitosan extracted from J + P (the combination that showed the highest yield) was analyzed at concentrations ranging from 0.25 mg/L to 2 mg/L. The antioxidant activity of the fungal chitosan increased from 64.05 ± 0.033% (at 0.25 mg/L) to 85.11 ± 0.024% (at 2 mg/L) (Figure 5). The IC_50_ value for the fungal chitosan was calculated to be 0.22 mg/L. The concentration-dependent increase in the percentage antioxidant activity of the extracted fungal chitosan can be attributed to the effect of nitrogen located at the C-2 locus of the chitosan structure [7].

Beyazit et al. [55] produced a chitosan Schiff base utilizing gossypol derived from cotton seeds. The antioxidant activity of the material was analyzed, revealing IC_50_ values of 16 and 12 μg/mL for the low- and high-molecular-weight chitosan synthesized materials, respectively. Savin et al. [56] extracted chitosan from the *Ganoderma lucidum* mushroom through chemical and enzymatic extraction methods. The IC_50_ value of the chemical extract and the enzymatic extract was found to be 67.61 ± 6.55 mg/mL and 2.02 ± 0.59 mg/mL, respectively, when subjected to antioxidant activity tests [56]. In another study conducted by Ai et al. [57], the antioxidant activity test for chitosan extracted from house flies (*Musca domestica*) resulted in an IC_50_ value of 0.373 mg/mL. *Tricholoma terreum* mushrooms were used by Koc et al. [40] to extract chitosan, which was then utilized to produce a film with a remarkable antioxidant activity of 84 ± 0.3%. The antioxidant activity values obtained in this study exceeded those reported in the literature. This finding expands the potential of chitosan as a food packaging material capable of protecting food materials from oxidative damage. The promising antioxidant activity of fungal chitosan extracted in this study revealed through the DPPH assay suggests that it can delay the autooxidation of foods wrapped with chitosan sheets, thereby enhancing the shelf-life of packed food. However, this needs further confirmation through extensive time-course studies in the future, as auto-oxidation can be affected by the type of food wrapped and other intrinsic and extrinsic factors.

### 3.4. Fabrication of Chitosan Sheet

**Figure 6:**
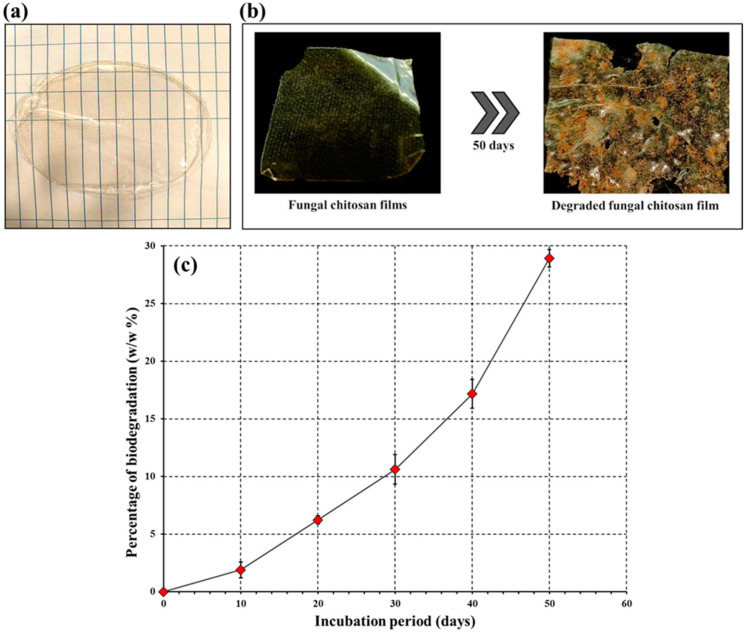
(**a**) Fabricated transparent fungal chitosan sheet, (**b**) biodegradation of chitosan sheets through soil burial, and (**c**) biodegradation profile of the fungal chitosan films.

The fabricated chitosan sheet appeared transparent, with a subtle yellow hue and texture resembling synthetic plastic sheets (Figure 6a). The surface appeared glossy and smooth with a uniform aesthetic appeal. The overall thickness of the films was 1 mm. The fungal chitosan film appeared similar to the synthesized chitosan sheet described by Zhang and Jiang [58]. A brown rice starch-based chitosan was created by Hasan et al. [59], which was similar to the film made in the current study using fungal chitosan.

### 3.5. Biodegradation Study of the Chitosan Film

The biodegradation of the fungal chitosan sheet spanning 50 d of soil burial is shown in Figure 6b. Biodegradation of a biopolymer involves sequential breakdown of the polymer into a simple compound through the effects of various microbial enzymes; hence, this process is called biodegradation [60]. Biodegradation commences with fragmentation of the biopolymer due to the combined effect of enzyme activity and physical factors. In the second phase, gradual depolymerization occurs with a decrease in polymer weight, and the monomers and degraded products resulting from depolymerization are taken up by microbes to carry out different metabolic processes [61]. Finally, in the last phase, complete mineralization occurs, involving the full oxidation of polymer residues [62]. The degradation rate of the biopolymer increased rapidly from the 10th day to the 50th day (Figure 6c). Biodegradation of the chitosan sheet increased steadily over the course of the soil burial study. After the 10th day, the biodegradation rate was 1.9 ± 0.69% (*w*/*w*), which then rose to 28.92 ± 0.75% (*w*/*w*) by the 50th day. A notable increase in biodegradation from 17.16 ± 1.25% (*w*/*w*) to 28.92 ± 0.75% (*w*/*w*) was observed between days 40 and 50, respectively. The slow fragmentation of the chitosan biopolymer could be related to the slow degradation rate up until the 10th day. The comparatively rapid biodegradation of the biopolymer sheet was observed until the 40th day, which can be attributed to the depolymerization of chitosan. The rapid weight reduction observed after the 40th day was likely due to the combined processes of depolymerization and mineralization. Visually, the degraded film surface appeared rough and eroded with pores and depressions, indicating colonization and microbial degradation [63]. Santhosh and Umesh [26] reported that the degradation of transparent food packaging plastic sheets was 7.89 ± 0.334 (*w*/*w*) after 20 d of soil burial. A report by Umesh et al. [50] stated that cellophane tapes showed a biodegradability of ~10% (*w*/*w*) after 4 weeks of soil burial. These reports suggest that the synthesized chitosan film has better biodegradability than plastic sheets. Pavoni et al. [64] subjected the modified chitosan film (with acetic acid and lactic acid) to soil burial to study the biodegradability. The results indicated that increased mechanical properties and structural breakdown started within the first 90 d of soil burial [64]. The brown rice starch-based chitosan nanocomposite film was subjected to a biodegradability test via soil burial. The results of the study indicated that, within 20 days of soil burial, the synthesized film underwent complete degradation, whereas the control chitosan film had minimal residue [59]. Deshmukh et al. [65] mixed chitosan with defatted *Chlorella* biomass to create composite films, which were later subjected to biodegradation studies. A 50% degradation was reported in a study of the composite film after 60 d of soil burial.

## 4. Conclusions

This study valorized agro-waste by converting it into value-added products, thereby reducing its environmental impact. *Aspergillus niger* DEL01, isolated from Pichavaram mangrove, was used for sustainable chitosan production. Replacing synthetic medium with pineapple peel waste significantly increased chitosan yield (139 ± 0.25 mg/L vs. 24 ± 0.8 mg/L). FTIR, XRD, TGA, and DSC confirmed its purity, crystallinity (51.61%), and thermal stability. Antioxidant activity (IC_50_ of 0.22 mg/L) and biodegradability (28.92 ± 0.75% after 50 days) were demonstrated. Chitosan films offer a biodegradable alternative to plastics, thereby reducing pollution. Further research could expand the applications by developing chitosan-based composites.

## Figures and Tables

**Table 1:** Comparison of chitosan yield.

Fungal Strain	Media	Yield
*Aspergillus niger* DEL01 (accession number: PP792611)	J (pineapple peel juice)	63.4 ± 0.17 mg/L
	J + P (pineapple peel juice + peptone)	139 ± 0.25 mg/L
	J + P + D (pineapple peel juice + peptone + dextrose)	98 ± 0.33 mg/L
	SDB	24 ± 0.8 mg/L

## Data Availability

Data are contained within the article and Supplementary Materials.

## Supplementary Materials

The following supporting information can be downloaded at: https://www.mdpi.com/article/10.3390/polym16172455/s1, Figure S1: Sample collection site at Pichavaram Mangrove, India; Figure S2: (a) DEL01 fungal plate (b) Microscopic structures of DEL01; Figure S3: (a) gDNA and ITS Amplicon QC data. (b) Phylogenetic Tree constructed with Sequences producing significant alignments. References [19,20,21,22,23,24,25,28] appear in the Supplementary Materials file.
