# Supplementary material for: Extraction of Fungal Chitosan by Leveraging Pineapple Peel Substrate for Sustainable Biopolymer Production
Source: Polymers (Basel). 2024 Aug 29;16(17):2455. doi: 10.3390/polym16172455 (PMC11397891; doi:10.3390/polym16172455)
Supplement: Supplementary file 1 [file polymers-16-02455-s001.zip › polymers-3099175-supplementary.pdf]

Supplementary

Extraction of Fungal Chitosan by Leveraging Pineapple Peel Substrate for Sustainable Biopolymer Production

Delwin Davis [1], Mridul Umesh [1], Adhithya Sankar Santhosh [1], Sreehari Suresh [1], Sabarathinam Shanmugam [2] and Timo Kikas [2,*]

[1] Department of Life Sciences, CHRIST (Deemed to be University), Hosur Road, Bengaluru 560029, Karnataka, India
[2] Institute of Forestry and Engineering, Estonian University of Life Sciences, Kreutzwaldi 56, 51014 Tartu, Estonia
* Correspondence: Timo.Kikas@emu.ee

Materials and methods

Isolation of fungus

Soil samples were collected from Pichavaram mangrove, Tamil Nadu, India (Figure. 1S) and stored at 4 °C. Subsequently, 1 g of soil was mixed with 100 mL autoclaved distilled water. The soil mixture was then serially diluted to 10^{-6} and the 10^{-4} and 10^{-6} dilutions were spread onto autoclaved Sabouraud dextrose agar (SDA) plates (dextrose, 40 g/L; peptone, 10 g/L; agar, 18 g/L) containing ampicillin antibiotic (0.1 g/L). This procedure was aimed at isolating fungi from the soil samples without bacterial contamination. The plates were incubated at room temperature for 7 days (Pradeep et al., 2013). The isolated fungal strains (sequentially labelled DEL01–DEL10) were subcultured in Sabouraud dextrose broth (SDB) (dextrose, 40 g/L; peptone, 10 g/L). Chitosan was extracted from all fungal isolate biomasses and the fungal isolate with the highest yield (DEL01) was selected. Macroscopic and microscopic observations of the DEL01 isolate were recorded after 7 days of incubation. Microscopic observation was performed after staining the fungal body with lactophenol cotton blue (LCB) stain. Cotton blue stains the cytoplasm, enhancing its visibility due to its dark blue coloration. Lactophenol serves as a clearing agent that helps to visualize fungal structures by clearing debris (Chatterjee and Das, 2020). Fungal inoculum discs of DEL01 were prepared by placing Whatman filter paper discs (5 mm diameter) on SDA plates and inoculating them with DEL01. This process ensured the uniform growth of the fungal mat on top of the paper discs.

Molecular identification for the fungal isolate

Microbial identification of the highest chitosan-yielding fungal isolate (DEL01) was performed using an ITS rRNA-based molecular method. Gene sequencing was performed using the Barcode Biosciences (Bangalore, Karnataka). A distance matrix and a phylogenetic tree were created using MEGA 10 (Umesh et al., 2023).

Results and discussion

Macroscopic and microscopic observations of DEL01

Pure fungal isolate plates from Pichavaram mangrove soil were subjected to macroscopic and microscopic analyses, followed by molecular characterization for species identification. Among all isolated fungi, DEL01 was selected for further study based on the yield of chitosan. Macroscopic characteristics for the current study included colony colour and texture, cleistothecia and sclerotia formations, the plate's reverse colour, exudate presence, and the production of soluble pigments [Figure. 2S(a)] (Samson and Varga, 2007). DEL01 exhibited a dark green colour with a greyish tint, attributed to the production of melanin pigment. It appeared velvety with dark rings forming as it grew. The sporing body appeared black and protruded from biomass. DEL01 displayed a dark brown to black coloration on the reverse side of the agar plates. Fungal microscopic analysis was performed after LCB staining (Nyongesa et al., 2015). The elongated conidiospores, characterized by a swollen vesicle on top, connected to a lengthy stalk known as the stipe, culminating in a brush-like arrangement called the metula housing phialides, signify the classification of the fungus within the *Aspergillus* genus [Figure. 2S(b)]. These conidiospores carry the asexual conidia.

Molecular characterization of DEL01

Fungal DNA was extracted and subjected to agarose gel electrophoresis, revealing the presence of a single band containing high-molecular-weight DNA [Figure. 3S(a)]. PCR amplification of fragments of the ITS region of the fungus resulted in the detection of a single distinct PCR amplicon band of ~600 base pairs upon resolution on an agarose gel. A forward and reverse DNA sequencing reaction of the purified PCR amplicon was performed using the BDT v3.1 Cycle sequencing kit on an AB |3730x| Genetic Analyzer. From the resulting reverse and forward sequences, a consensus sequence of the PCR amplicon was generated using the Aligner software. The generated ITS sequence was analyzed using BLAST against the NCBI GenBank database to determine the genetic identity of the species. First ten sequences of the BLAST results were selected based on the maximum identity score for alignment using Clustal W (multiple alignment software program). Finally, using MEGA 10 software, a phylogenetic tree of the selected sequence and the query sequence was constructed based on nucleotide homology and phylogenetic analysis [Figure. 3S(b)]. It was found out that the fungal strain to be *Aspergillus niger*. The obtained sequence was later submitted to GenBank; hence, the organism of the current study was *Aspergillus niger* DEL01 under the accession number PP792611. *Aspergillus* spp. has been isolated from mangrove soil for polyethylene bag degradation (Eldin et al., 2022). In a study conducted on Pichavaram mangrove soil sediment, four different species of *Aspergillus* were identified. This study aimed to investigate the interactions between microbiomes in natural habitats (Manivel et al., 2020).

Figure S1. Sample collection site at Pichavaram Mangrove, India

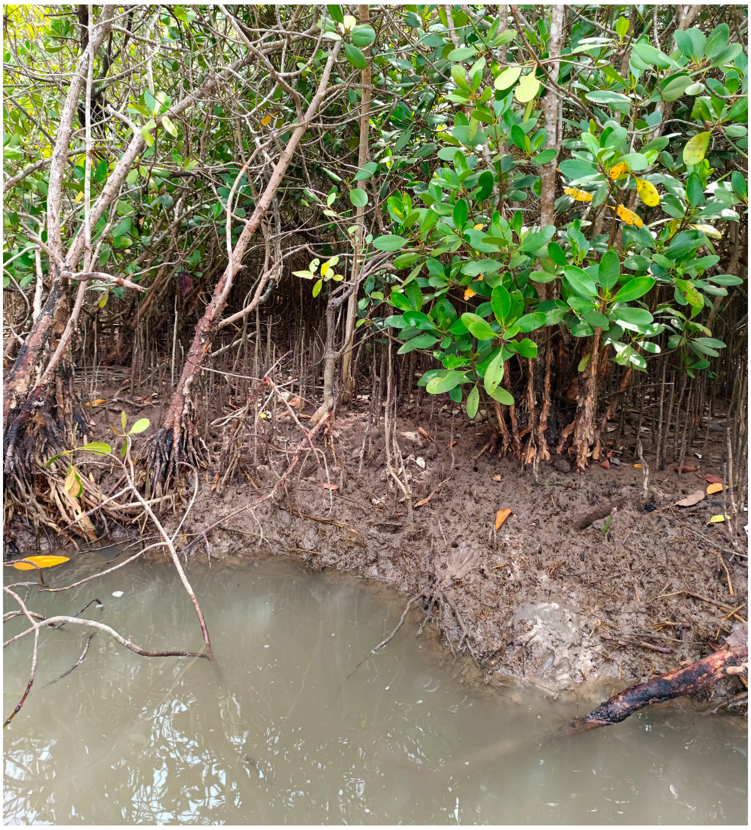

Figure S2. (a) DEL01 fungal plate (b) Microscopic structures of DEL01.

(a)

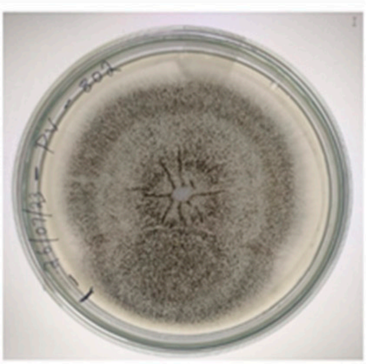

(b)

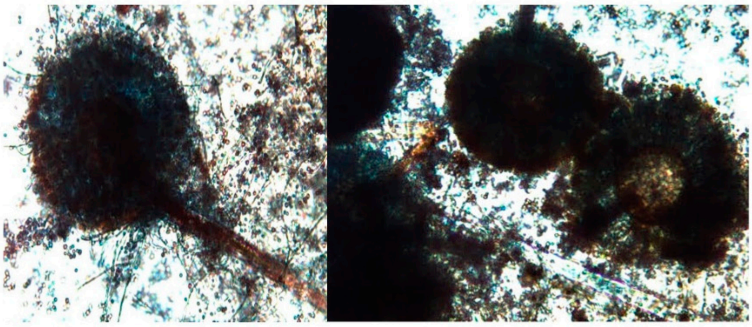

Figure S3. (a) gDNA and ITS Amplicon QC data. (b) Phylogenetic Tree constructed with Sequences producing significant alignments

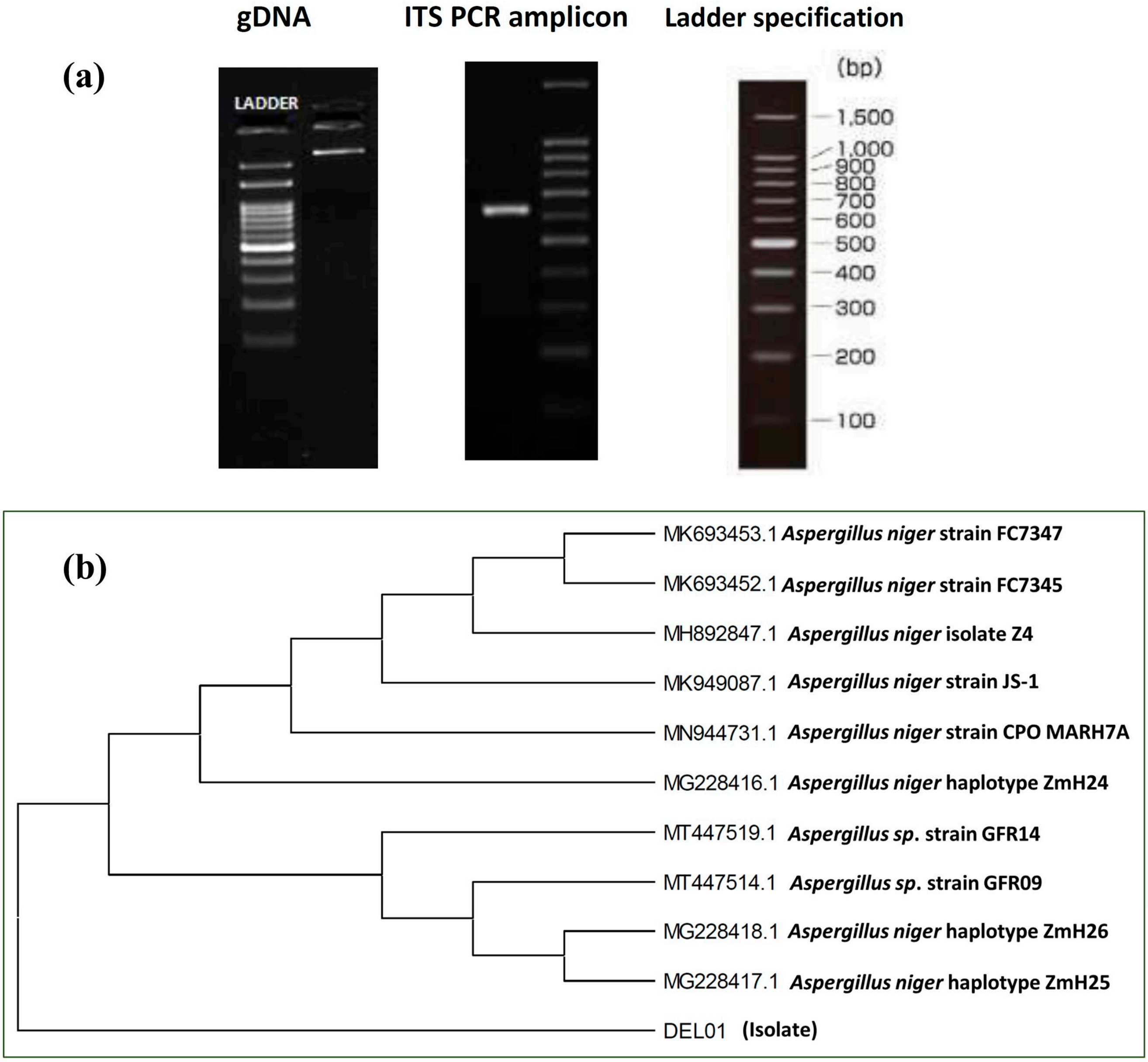

Reference

1. Pradeep, F.S., Begam, M., Palaniswamy, M., Pradeep, B. 2013. Influence of culture media on growth and pigment production by *Fusarium moniliforme* KUMBF1201 isolated from paddy field soil. *World Appl. Sci.,* J. **22** (1): 70-77, 2013.
2. Chatterjee, S., Das, S., 2020. Developmental stages of biofilm and characterization of extracellular matrix of manglicolous fungus *Aspergillus niger* BSC-1. *J. Basic Microbiol.,* **60**(3), 231-242.
3. Cicciù, M., Fiorillo, L., Cervino, G. 2019. Chitosan Use in Dentistry: A Systematic Review of Recent Clinical Studies. *Mar. Drugs,* **17**(7).
4. Umesh, M.; Suresh, S.; Santosh, A. S.; Prasad, S.; Chinnathambi, A.; Al Obaid, S.; Jhanani, G. K.; Shanmugam, S. 2023. Valorization of pineapple peel waste for fungal pigment production using *Talaromyces albobiverticillius*: Insights into antibacterial, antioxidant and textile dyeing properties. *Environ. Res.*, 229, 115973.
5. Samson, R.A., Varga, J. 2007. *Aspergillus* Systematics in the Genomic Era. *CBS Fungal Biodiversity Centre*.
6. Nyongesa, B.W., Okoth, S., Ayugi, V. 2015. Identification Key for *Aspergillus* Species Isolated from Maize and Soil of Nandi County, Kenya. *Adv Microbiol*.
7. Mohy Eldin, A., Al-Sharnouby, S.F.S., ElGabry, K.I.M., Ramadan, A.I. 2022. *Aspergillus terreus*, *Penicillium* sp. and *Bacillus* sp. isolated from mangrove soil having laccase and peroxidase role in depolymerization of polyethylene bags. *Process Biochem.,* 118, 215-226.
8. Manivel, G., Raj, D.M.L., Prathiviraj, R., Senthilraja, P. 2020. Distribution of phylogenetic proximity upon species-rich marine Ascomycetes with reference to Pichavaram mangrove soil sediment of southern India. *Gene Reports,* 21, 100878.
